# Endosomal crosstalk: meeting points for signaling pathways

**DOI:** 10.1016/j.tcb.2012.06.004

**Published:** 2012-09

**Authors:** Máté Pálfy, Attila Reményi, Tamás Korcsmáros

**Affiliations:** 1 Department of Genetics, Eötvös Loránd University, Pázmány P. s. 1/C, Budapest, H-1117, Hungary; 2 Department of Biochemistry, Eötvös Loránd University, Pázmány P. s. 1/C, Budapest, H-1117, Hungary; 3 Department of Medical Chemistry, Semmelweis University, Tűzoltó u. 37-47, Budapest, H-1094, Hungary

## Abstract

Endocytosis participates in downregulating incoming signals, but ‘signaling endosomes’ may also serve as physical platforms for crosstalk between signaling pathways. Here, we briefly review the role of endosomes in signaling crosstalk and suggest that endosome-associated scaffold proteins mediate this crosstalk. In addition, using a proteome-wide *in silico* approach – in which we analyze endosome-binding properties and the capacity of candidates to recruit signaling proteins from more than one distinct pathway – we extend the list of putative crosstalk-mediating endosomal scaffolds. Because endosomal crosstalk may be an important systems-level regulator of pathway communication, scaffold proteins that mediate this crosstalk could be potential targets for pharmacological intervention and synthetic engineering.

## Endosomes

Endocytosis is a cellular process by which cell surface components and extracellular molecules are internalized into lipid vesicles called endosomes (Box 1). Endocytosis has long been considered an effective mechanism to downregulate cellular signaling events by internalizing receptors or ligand–receptor complexes [1,2]. However, increasing evidence suggests that endocytosis can also contribute actively to signaling, which has led to the signaling endosome hypothesis [3–6]. Endosomes can regulate the localization of signaling complexes either by spatially restricting signaling activity to particular loci in the cell or by acting as vesicular carriers, propelled by molecular motors, to transport signaling proteins to cellular locations that are unreachable by diffusion [7]. Endosomes can also isolate signaling components and prevent unwanted signaling interactions. This strategy is likely to occur in the regulation of, for example, glycogen synthase 3 beta (GSK3-β), a promiscuous kinase that has numerous phosphorylation targets in distinct pathways, including WNT, Hedgehog, epidermal growth factor (EGF)/mitogen-activated protein kinase (MAPK), and transforming growth factor beta (TGF-β) signaling. Sequestering GSK3-β into endosomes plays an essential role in the WNT pathway in *Xenopus*, because this step prevents the phosphorylation and subsequent proteasomal degradation of β-catenin, a key component of the pathway [8]. Recently, it was suggested that imprisoning GSK3-β may also be essential for insulating it from other signaling pathways such as Hedgehog or TGF-β, thereby preventing the phosphorylation and subsequent degradation of specific signaling components [9].

A signaling endosome also serves as a ‘meeting ground’ for signaling components [10]. Signaling enzymes or other signaling effectors such as GTPases and their regulators or even transcription factors are often specifically localized to endosomes through endosome-associated adaptor and scaffold proteins (see Glossary). Thus, the endosome membrane facing the cytoplasm forms a physical platform for signaling complex assemblies where endosomal scaffolds can facilitate signaling reactions between the recruited components, similarly to the general role of scaffolds in the plasma membrane and in the cytoplasm [11]. For example, in the EGF/MAPK pathway, the MP1–p14 scaffold complex, anchored to the late endosomal membrane by the p18 protein [12], localizes MEK1 to late endosomes and promotes the phosphorylation of ERK1 and ERK2 kinases [13]. The MP1/p14/p18 scaffold complex is also known as the LAMTOR1-3 complex. A recent proteomic study in *p14*^−*/*−^ mouse embryonic fibroblasts identified 31 proteins whose regulation was p14 dependent [14]. In addition, the MP1/p14/p18 scaffold complex recruits the mTORC1 complex via Rag GTPase proteins to lysosomes on stimulation with amino acids. This ‘Ragulator’ complex enables activation of mTOR by its lysosomal activator, the Rheb GTPase in human cells [15]. Another example for the role of endosomal scaffolds is SARA, an endosome-specific scaffold that can enhance TGF-β signaling by bringing the TGF-β receptor and its phosphorylation targets, SMAD2 and SMAD3, into close proximity, as shown in cultured hamster cells [16]. Likewise, a further endosome-specific adaptor, Endofin, facilitates SMAD4 phosphorylation in human cell culture [17].

**Figure 1:**
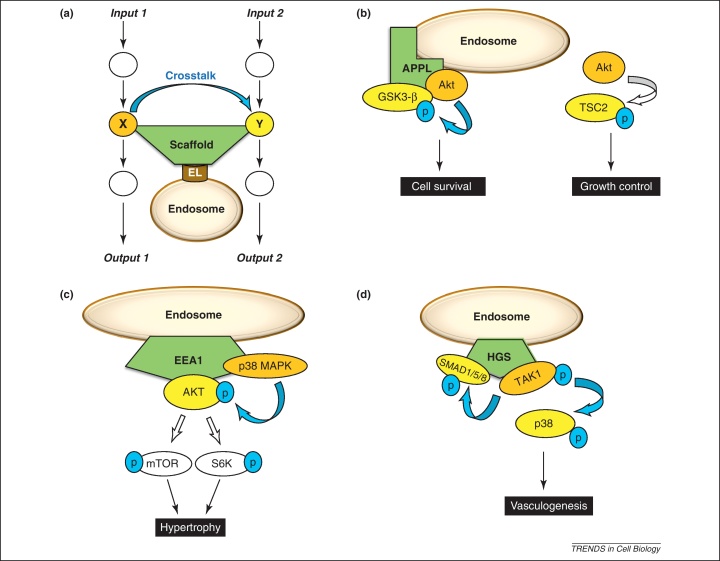
**Endosome-associated adaptors/scaffolds mediate signaling specificity, localization, and crosstalk**. Green color shows scaffold proteins; orange and yellow shows scaffold-interacting proteins; brown shows endosome-related proteins; blue arrows represent crosstalk; white arrows represent other post-translational modifications; black squares represent output functions. (**a**) Crosstalk between two pathways may be localized to endosomes as physical platforms through crosstalk mediating scaffolds that bind to the endosome. EL denotes Endosome Localization domains (Box 1). (**b**) In zebrafish embryos, the endosomal adaptor APPL mediates crosstalk between glycogen synthase 3 beta (GSK3-β) and AKT, but is not required for TSC2 activation by AKT. (**c**) On angiotensin-II signaling, early endosome antigen 1 (EEA1) recruits signaling components to endosomes and mediates the crosstalk between p38 and AKT. (**d**) In bone morphogenetic protein (BMP) signaling, the endosomal scaffold hepatocyte growth factor-regulated tyrosine kinase substrate (HGS) facilitates the crosstalk between SMADs and the TAK1 kinase. This phosphorylation event is HGS dependent.

Scaffold proteins can not only facilitate signal transduction within a pathway, but are also known to regulate interactions between different signaling pathways at the plasma membrane or in the cytoplasm [18]. This type of interaction, where the interacting proteins function in distinct signaling pathways, is generally referred to as ‘crosstalk’ [19]. In many species, the scaffold protein AXIN mediates crosstalk between multiple pathways in the cytoplasm [20]. For instance, different WNT pathway components (e.g., DVL, CKIɛ, GSK3-β) have been shown to modulate the activation of JNK and p38 cascades in the EGF/MAPK pathway through interacting with AXIN [20,21]. Interestingly, on WNT-induced signaling, GSK3-β, DVL, and AXIN have been shown to localize to the cell membrane as well as to endosomes following internalization of the β-catenin destruction complex [22,23]. AXIN also can integrate TGF-β and WNT signals; it functions as a negative regulator of WNT and a positive regulator of TGF-β pathways [24]. Recent studies highlighted some of the scaffolds that appear to mediate crosstalk on the surface of endosomes. For example, the endosomal scaffold protein APPL can facilitate crosstalk between AKT and GSK3-β kinases, which are key components of insulin and WNT pathways, respectively [25]. The endosomal scaffold EEA1 can mediate crosstalk between EGF/MAPK and insulin pathways by connecting p38 and AKT [26]. Hepatocyte growth factor-regulated tyrosine kinase substrate (HGS), another endosomal scaffold, was recently shown to mediate crosstalk between the TGF-β and EGF/MAPK pathways [27]. Here, using an *in silico* approach, we propose additional scaffolds that have the potential to mediate crosstalk on endosomes, and suggest that these endosomes can serve as a physical platform specifically to mediate signaling crosstalk (Figure 1a).

## Signaling crosstalk

In general, signaling crosstalk is an interaction between components of multiple signaling pathways. Here, we define ‘crosstalk’ as a physical interaction between proteins of two (or more) distinct signaling pathways. However, we note that, in genetics, the term crosstalk is sometimes used to denote transcriptional connections between genes functioning in different pathways. Although historically pathways have been viewed as discretely linear, with the advent of network biology it became evident that these pathways are densely interconnected via signaling crosstalk. The importance of crosstalk is apparent if we consider that, in contrast to the wide variety of signaling functions and the macroscopic and microscopic diversity of living forms, the number of signaling pathway types is relatively low (a few dozen) [28,29]. Because the number and combinations of transduceable signals are limited, crosstalk between pathways can create novel input/output combinations. Having more input/output combinations increases the possible ways that signaling information can flow within the cell, which contributes to allowing more diverse phenotypes. Thus, crosstalk plays an important role in, for example, developmental processes, regeneration, immune response, and stress adaptation [30–33]. Malfunction of crosstalking proteins (e.g., IRS1, JNK1) can cause major systems-level diseases, such as cancer or diabetes [34,35]. During tumorigenesis, for instance, rewiring of signaling networks is achieved by the alteration of crosstalking proteins (e.g., change in ERK–GSK3-β crosstalk) [34,36,37]. Consequently, pharmacological targeting of crosstalking proteins could be an important strategy in the future [38–40].

Previously, we examined eight biochemically and evolutionarily defined signaling pathways important in development (EGF/MAPK, insulin, TGF-β, Notch, WNT, Hedgehog, JAK/STAT, and NHR pathways) and found that crosstalk can occur between any two of these pathways [40]. Thus, theoretically, all of these pathways can influence each other. This phenomenon, which is supported by experimental observations of the high number of crosstalk possibilities within the insulin pathway, raises serious regulatory problems for the cell [35]. Thus, crosstalk must be precisely regulated to prevent ‘leaking’ or ‘spillover’ [41]. This can be achieved using different insulating mechanisms; for example, via scaffolding proteins or compartmentalized reactions [18,41]. Here, we suggest endosomes as one means to facilitate crosstalk (Figure 1a).

## Crosstalk mediated by endosomal scaffolds

Considering the known dynamic and spatial role of endosomes in signaling, we argue that those endosomes that possess scaffolds capable of connecting proteins from different pathways can localize, isolate, and modulate pathway interactions. Accordingly, we term these endosomes ‘crosstalk endosomes’. Scaffold proteins can be recruited to these endosomes by binding to endosome-specific phosphoinositides (PtdIns) and endosome-related proteins (Box 1) [42]. Different PtdIns lipid compositions enable the specific binding of proteins through specific protein domains (Box 1). In the following, we present three endosome-associated scaffolds with known roles in mediating crosstalk.

The earliest group of early endosomes is characterized by the scaffold protein APPL. Numerous studies in recent years have provided striking examples of how endosomally localized APPL regulates the crosstalk specificity of AKT. For example, APPL is important in crosstalk between the insulin and WNT signaling pathways because it interacts directly with AKT (an insulin pathway member) and GSK3-β (a WNT pathway member) on endosomes [25]. AKT is a signaling hub and regulates various cellular functions such as cell survival, growth, proliferation, and metabolism; thus, its output signals must be specifically regulated. The role of APPL in AKT signaling in zebrafish embryos was examined and revealed that endosomal APPL1 is required for specific mediation of the phosphorylation of GSK3-β, but not of other substrates of AKT, during development [25] (Figure 1b). Similarly, APPL1–AKT signaling was specifically required for survival of stomach/duodenum and pancreas progenitor cells in *Xenopus laevis* embryos [43]. In addition, the crosstalk-mediating role of APPL was recently identified in adiponectin signaling, although we note that this crosstalk has not been explicitly described as localized to endosomes [44]. Here, APPL can simultaneously bind the TGF-β pathway member TAK1 and the EGF/MAPK member MKK3 to facilitate p38 activation and specify adiponectin signaling [44].

In canonical EEA1 early endosomes, a novel function of EEA1 is as a scaffold in angiotensin-II induced AKT activation, which induces hypertrophy of vascular smooth muscle cells [26]. On angiotensin-II stimulation, colocalization and immunoprecipitation experiments showed that AKT interacts with EEA1, which promotes its phosphorylation by recruiting kinases such as p38 of the EGF/MAPK pathway [26] (Figure 1c). Because the downregulation of EEA1 inhibited AKT phosphorylation [26], we assume that EEA1 is important for mediating this crosstalk between the EGF/MAPK and insulin pathways [26].

The HGS (HRS) scaffold protein has recently been shown to play a role in bone morphogenetic protein (BMP) signaling (a specific subtype of the TGF-β pathway) during mouse embryogenesis [27]. Signal transduction downstream of BMP receptors occurs mainly by SMAD proteins that form complexes and transmit signals to the nucleus. In vertebrate embryogenesis, in addition to SMAD signaling, TAK1/p38 phosphorylation is also required to transduce BMP signals [27]. TAK1 serves as a multi-pathway protein that transduces the BMP signal to the MAPK signaling pathway [45]. HGS is proposed to play a key role in simultaneously promoting TAK1/p38 and SMAD phosphorylation by scaffolding the TAK1 and SMAD1/5/8 complexes on endosomes [27] (Figure 1d).

Given these examples and the findings presented above, we propose that the major mediators of crosstalk on endosomes are the endosome-associated scaffold proteins. Are there more crosstalk-mediating scaffolds on endosomes awaiting discovery?

## Additional endosomal scaffolds: do they also mediate crosstalk?

Based on the presented examples, we collected information on domain compositions, protein interactions, and pathway memberships (Table S1 in the supplementary material online) to identify scaffold proteins capable of mediating crosstalk on endosomes (Box 2). There are three ways a scaffold protein can be endosome associated: (i) it binds the endosomal membrane; (ii) it binds an endosomal protein; or (iii) it binds another scaffold that is already bound to the endosome (i.e., indirect or scaffold complex member). Accordingly, we found and classified 76 endosome-associated scaffolds based on their binding properties (see Figure I in Box 2).

Next, we examined the pathway memberships of signaling proteins interacting with endosome-associated scaffolds to select those that connect interacting proteins from more than one pathway (Box 2) and identified 49 potential crosstalk-mediating endosomal scaffolds. (In Table S2a in the supplementary material online, we list these scaffolds, their endosome association type and the signaling pathways to which these scaffolds are directly connected. In Table S2b in the supplementary material online, all supporting protein–protein interaction network data are presented, showing the interactions between the endosomal scaffolds and their partner proteins from different pathways. Note that the partner proteins of a scaffold are interacting (crosstalking); thus, a scaffold and its partners form an interaction triangle.) Interestingly, among these 49 crosstalk-mediating endosomal scaffolds, we found four (GRB1, CCNE1, SARA, AXIN1) that were potentially capable of connecting proteins from five different pathways (JAK/STAT, Notch, WNT, TGF-β, and the RTK pathway, which contains insulin and EGF/MAPK cascades), indicating that endosomal crosstalk via these scaffolds can have important systems-level effects (Table 1a subset of Table S2b in the supplementary material online).

## Two examples: AXIN and SARA

We briefly present two scaffolds, AXIN and SARA, which were found in our analysis and could potentially be mediators of crosstalk on endosomes. Both are known scaffolds, but a crosstalk-mediating function is known only for AXIN, whereas endosomal localization is known only for SARA. Based on our compilation, we hypothesize that both scaffolds can be localized on endosomes and may mediate crosstalk by interacting directly with components of multiple pathways.

**Figure 2:**
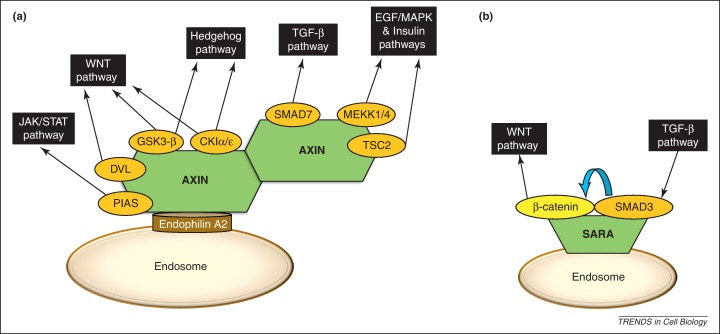
**Two scaffolds that could potentially mediate crosstalk on endosomes**. **(a)** A hypothetical arrangement showing AXIN as an endosomal scaffold able to connect multiple pathways (not all known AXIN binding proteins are shown). Based on the AXIN binding partners from [20] and the interaction between AXIN and the endosomal adaptor Endophilin A2 [50]. (**b**) A predicted picture of the β-catenin–SMAD3 interaction mediated by the endosome-bound SARA.

AXIN was previously described as a master scaffold for multiple signaling pathways [20]. Besides its distinct roles in the WNT, TGF-β, and EGF/MAPK (p38 and JNK) pathways, AXIN has integrative and crosstalk functions [20]. Until now, however, AXIN had not been shown to be associated with endosomes. Using the HPRD, BioGRID, and STRING protein–protein interaction resources, we examined AXIN interactor proteins and selected those that are known to localize at the endosome based on their Gene Ontology annotation [46–49]. This analysis indicated a yeast two-hybrid screen [50] that shows with medium confidence that AXIN can bind Endophilin A2 (encoded by *SH3GL1*), an SH3-domain-containing endosomal protein. In addition to the role of Endophilin family members in the formation of endocytic vesicles, they may also interact with signaling proteins [51–53]. The interaction of Endophilin A2 with AXIN may allow this latter multi-pathway scaffold to be localized to endosomes and thus the endosome membrane could serve as a physical platform for AXIN-mediated pathway crosstalk (Figure 2a). We note that many of the assumptions of this model, which is based on data mining, need to be explicitly tested experimentally. We also note, however, that AXIN has recently been proposed to localize to endosomes through an internalized WNT receptor complex [9].

As described above, SARA is an endosome-specific scaffold important in TGF-β signaling because it binds SMAD2 and SMAD3 [16]. However, a yeast two-hybrid screen in mammalian cells showed that SARA also binds β-catenin, a key protein of the WNT pathway [54]**,** but the functional role of this interaction has not been identified. β-catenin had already been found in endosomal fractions in human embryonic kidney cells and was associated with another endosomal scaffold, APPL1 [55,56], suggesting that endosomes could modulate β-catenin signaling. Moreover, in COS cells (kidney cells, African green monkey) SMAD3 was found to interact with β-catenin, resulting in increased protein stability by protecting β-catenin from degradation and increased transcriptional activity by facilitating the nuclear translocation of β-catenin during chondrogenesis [57]. Together, these observations suggest that endosomally localized SARA may connect SMAD3 and β-catenin and affect the dynamics of the TGF-β and WNT pathways. Colocalization of SMAD3 and β-catenin on the endosome-bound SARA scaffold may alter the cytoplasmic degradation rate of β-catenin such that it could more efficiently enter the nucleus (Figure 2b). Again, we note that the colocalization of SMAD3 and β-catenin on endosomes must be experimentally validated and the crosstalk-facilitating function of SARA also needs to be explicitly tested.

## Engineering synthetic endosomal crosstalk

It well established that, for a systems-level understanding of signaling network behaviors, one needs to address the dynamic and spatial aspect of cellular signaling to understand how information is dissipated from the cell membrane across the cytoplasm towards the nucleus [58]. Therefore, similar to how researchers previously made great advances in controlling the dynamic aspect of individual signaling pathway outputs by using artificially modified natural protein scaffolds [59], we propose that the endosomal membrane facing the cytoplasm could be an invaluable physical platform to facilitate crosstalk in a spatially controlled fashion. Endosomes traverse the cell as they move inwards from the cell membrane toward the nucleus and their outer membrane is a unique surface to initiate artificial connections between signaling components.

The reconstruction-based approaches of synthetic biology could be used, for example, to modify natural scaffolds to enable them to form novel protein–protein or protein–lipid interactions and observe whether this changes their crosstalk activity [60]. The localization of a protein scaffold directly or indirectly to endosomes may be mediated by endosomal membrane-interacting domains. These domains bind to distinct phosphoinositide species, which are characteristic of the membranes of different endosome populations; for example, the PX domain binds PtdIns(3)P (Box 1). Thus, the capacity of scaffolds to facilitate physical interactions between two proteins, and the ability of endosomal membranes to act as physical dynamic platforms, can be united to colocalize crosstalk-mediating agents in time and space [11,61]. This strategy may be useful for probing the significance of different crosstalk mechanisms relying on the spatial aspect of intracellular signaling (i.e., the influence of the spatial localization of signaling complexes on the activity of a pathway interaction).

The EGF receptor is an example of a signaling component known to yield different outcomes based on the spatial localization of its activity. Signals emanating from internalized EGF receptors located on the endosome membrane en route to an internal destination can have different outcomes compared with signals initiated by activated EGF receptors at the plasma membrane [62,63]. Therefore, because of the numerous deactivating mechanisms (e.g., phosphatase activity on protein kinases) and the long distances that signals travel within the cell – especially in highly polarized cells such as neurons with long axons – endosomes that are actively moved across the cytoplasm could play an essential role in ensuring that weak crosstalk signals eventually exert their effect [64]. In some instances, spatial localization of active signaling receptors between the plasma membrane and the endosome can result in opposite physiological outputs. For example, on stimulation, the tumor necrosis factor (TNF) receptor-1 signals from the plasma membrane and promotes survival via nuclear factor kappa B (NFκB) [65]. However, internalization of TNF receptor-1 to endosomes disables NFκB activation and stimulates caspase-8, leading to apoptotic cell death. Thus, internalization and altered localization of the signaling complex provide a key switch mechanism between two different signaling responses [65]. Speculatively, the endosome membrane may be exploited as a spatial ruler of distance between the plasma membrane and the nucleus, because the lipid and protein composition of the endosomal membrane changes as the endosome moves inward from the early endosomal populations near the plasma membrane to late endosomes near the nucleus (see Figure I in Box 1). Early versus late endosomal membrane-binding synthetic scaffolds could be used to physically link artificially modified/designed signaling components (e.g., kinases/phosphatase, GTPases, proteases) at different distances along the plasma membrane–cytoplasm axis.

More specifically, transcription factors, which are normally the terminal players in signaling pathways, may utilize distance-based control to modulate the strength and duration of the signal and communication with other pathways; transcription factors activated far from the nucleus (e.g., at early endosomes) may be inactivated before reaching the nucleus, whereas those that are activated in close proximity to the nucleus (e.g., at late endosomes) have a better chance of entering the nucleus in their active form. Thus, an important function of endosomes may be the spatiotemporal regulation of transcription factor activation. For instance, on interleukin (IL)-6 induction, endosome-mediated signaling crosstalk can occur between ERK (EGF/MAPK pathway) and STAT3 (JAK/STAT pathway), which facilitates STAT3 interaction with its transcriptional co-activators CBP and p300 [66]. Because ERK1/2 phosphorylation occurs in late endosomal structures, which localize close to the nucleus, this endosome-associated crosstalk maximizes the transcriptional activity of STAT3 [66]. Furthermore, the distance of STAT3-containing endosomes (often called sequestering endosomes) from the nucleus could determine the duration of the signal [67,68]. Endosomes may also aid in the transport of weak signals, exemplified by HGF-induced cMet signaling, where endosomal trafficking is required for the STAT3 signal to reach the nucleus, in contrast to the strong ERK1/2 signal which is not dependent on endocytic trafficking [69]. Similarly, late endosomal structures (i.e., multivesicular bodies) play a positive role in NFκB signaling by sequestering IκB (inhibitor of NFκB) in the *Drosophila* Toll pathway [9,70]. *In vivo* genetic experiments in *Drosophila* have confirmed that endocytic trafficking modulates the strength of the transcriptional signal [71,72]. Therefore, with synthetic manipulation of transcription factor–endosome associations, we could potentially modulate and specifically facilitate the spatial properties, activity, and possible interactions of transcription factors, such as STAT3 or NFκB.

## Concluding remarks

Specificity, strength, and localization are key properties in interpathway communication. Their precise regulation can be maintained by signaling endosomes serving as physical platforms for signaling pathway crosstalk. Based on this, we coined the term ‘crosstalk endosome’ and assume that endosome-associated scaffold proteins may be the principle components mediating the interaction of different pathway components. Regulating the expression of endosomal scaffolds is likely to be an important mechanism for cells to control signal transduction pathways. Therefore, mutation or changes in the expression of endosomal scaffolds may have pathological effects, as demonstrated in a newly identified primary immunodeficiency syndrome in which a point mutation in the 3′ untranslated region (UTR) of the *p14* gene resulted in decreased expression of the p14 late endosomal scaffold [73]. Impairment of retrograde neurotrophin signaling is likely to cause ‘vesicular traffic jams’ that have been linked to various neurodegenerative diseases. Indeed, accumulation of the Aβ42 protein in early endosomes is a feature of Alzheimer's disease, and TrkA signaling and transport are dependent on huntingtin-associated protein 1, implicating that defects in endosomal signaling contribute to the development of neurodegenerative diseases [74]. Despite their key regulatory role, there are only a few dozen genetic alterations of endocytic genes that have been linked to malignancies [75]. Among them, overexpression of Rab5a, a canonical marker of early endosomes, is observable in lung cancer and hepatocellular carcinoma [76,77]. Also, HGS and other components of the endosomal sorting complex required for transport (ESCRT) machinery are misregulated in various cancers [78].

Future studies will reveal whether endosomal crosstalk is a widespread phenomenon or applies to only a limited number of signaling systems. It is intriguing to speculate that ‘crosstalk endosomes’ may constitute a specialized population of endosomes; however, detecting the dynamics and signaling effects of crosstalk endosomes faces several technical challenges (Box 3). Engineering synthetic endosomal crosstalk may be one useful way of testing the relevance of this phenomenon. If our assessment is correct, the next few years will see the clarification of crosstalk mechanisms, the experimental discovery of more crosstalk mediating scaffolds, and illumination of the spatial aspect of intracellular signaling – particularly the role of endosomes in this process.

## Figures and Tables

**Figure I:**
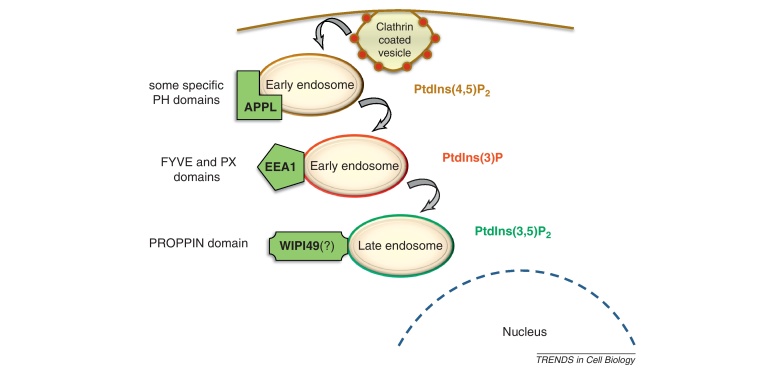
**Phosphoinositide-specific binding of proteins to distinct endosomal populations**. The change of phosphoinositides (PtdIns) on endosomes is reflected in their protein composition, because their associated protein factors often bind to endosomal membranes via modular domains (e.g., PH, FYVE, PX, or PROPPIN) that can specifically interact with distinct forms of PtdIns. Note that WIPI49 is one of the few PROPPIN domain-containing proteins and we hypothesize that it could bind to the membrane of late endosomes, though experimental validation is needed to prove its late endosomal localization.

**Figure I:**
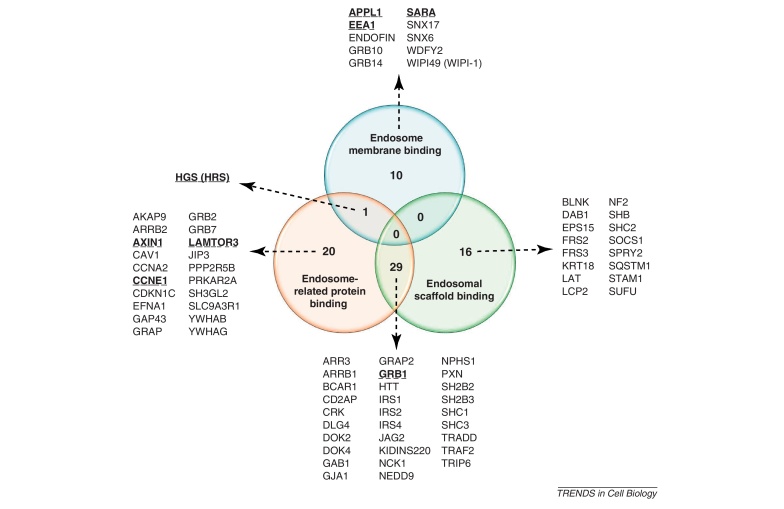
**Overlapping classes and lists of the 76 endosome-associated scaffolds based on their binding properties**. In humans, we identified 10 scaffolds that can bind to endosomal membrane (blue), 20 scaffolds that bind to endosome-related proteins (red), and 16 indirect endosomal scaffolds that interact with directly binding endosomal scaffolds (green). In addition, 29 scaffolds can bind both to endosome-related proteins and to directly binding endosomal scaffolds (red–green overlap). Only one scaffold hepatocyte growth factor-regulated tyrosine kinase substrate (HGS) can bind both the endosomal membrane and endosome-related proteins (blue–red overlap). Scaffolds that are mentioned in the main text are highlighted.

**Table 1:** Selected examples of predicted endosomal scaffolds and their crosstalking protein partners

Scaffold^a^	Scaffold partner protein 1		Scaffold partner protein 2	
Name	Pathway	Name	Pathway
AXIN1	TSC2	RTK	PPP2CA	TGF, WNT
TSC2	RTK	SMAD2	TGF, WNT
CTNNB	NOTCH, WNT	SMAD7	TGF
DVL1	NOTCH, WNT	SMAD7	TGF
PIAS1	NOTCH, JAK/STAT	SMAD7	TGF
CCNE1	RB2	TGF	CCND3	WNT, JAK/STAT
RBL1	TGF	HDAC1	NOTCH
RB2	TGF	HDAC1	NOTCH
BRCA1	NOTCH	E2F4	TGF
CCND3	WNT, JAK/STAT	CDKN1B	RTK
GRB1	CTNNB	NOTCH, WNT	FLT	JAK/STAT
INSR	RTK	VAV	NOTCH, JAK/STAT
JAK2	JAK/STAT	ABL	NOTCH, RTK
EGFR	RTK, JAK/STAT	CTNNB	NOTCH, WNT
50 more crosstalking partners for GRB1 are found in Table S2b in the supplementary material online			
SARA	ALK5	TGF, RTK, JAK/STAT	CTNNB	NOTCH, WNT
CTNNB	NOTCH, WNT	SMAD7	TGF
ALK5	TGF, RTK, JAK/STAT	MYL	NOTCH

a For the complete list of the 49 predicted endosomal scaffolds able to mediate crosstalk, see Table S2b in the supplementary material online, where the proteins also contain hyperlinked UniProt ACs.

## Box 1 The endocytotic pathway

Endocytosis, the cellular process of internalizing transmembrane receptors and extracellular cargo, occurs through many routes. The most extensively studied endocytic pathway is clathrin-mediated endocytosis, in which internalization is mediated by the formation of clathrin-coated pits that bud off to form clathrin-coated vesicles (CCVs). Receptors can also be brought to the inside of the cell by myriad clathrin-independent mechanisms [79]. After the dissociation of the coat, vesicles form the early endosome, which is the primary sorting station along the endocytic pathway. By pinching off from the elongated tubules of the early endosome, recycling endosomes can carry the internalized receptors back to the plasma membrane. However, ubiquitylated receptors are recognized by the early ESCRT, which sorts receptors to the late endosomal/multivesicular body compartment that ultimately fuses with lysosomes, resulting in the degradation of receptors.

RAB proteins and PtdIns species are the main molecular signs that define the identity of different endosomal compartments (Figure I). They can easily be modified by different proteins, including PtdIns kinases and phosphatases, allowing the dynamic control of endosomal spatial distribution [42]. The plasma membrane is characterized by PtdIns(4,5)P_2_, which has an important role in forming clathrin-coated vesicles through its specific interaction with AP-2. After pinching off the plasma membrane, the vesicles start to increase in PtdIns(3)P content, a characteristic of early endosomes. Through the action of phosphoinositide kinases, multivesicular bodies/late endosomes become enriched in PtdIns(3,5)P_2_. Through specific protein domains, these PtdIns species enable the specific binding of proteins, including adaptors and scaffolds. Binding to PtdIns(3)P occurs through FYVE domains and PX domains, while PH domains may interact with PtdIns(3)P on endosomes as well as with PtdIns(4,5)P_2_, PtdIns(3,4)P_2_, and PtdIns(3,4,5)P_3_ regions of the plasma membrane [80]. Binding to PtdIns(3,5)P_2_ can be achieved via the PROPPIN domain [81]. Similarly, RAB proteins bind to their specific effector proteins, but the PtdIns content of the membrane can influence the affinity of their binding.

## Box 2 Identification of crosstalk mediating endosomal scaffolds

To list scaffold proteins capable of mediating crosstalk on endosomes, we used a list of known and potential scaffolds from [82]**,** a list of endosome-related proteins based on Gene Ontology cellular compartment annotation [47]**,** and a list of endosomal membrane-binding domains from [80].

First, we used the PFAM protein domain resource [83] to select scaffolds that contain endosomal membrane (phosphoinositide–lipid) binding domains (FYVE, PX, PH, PROPPIN domains) (listed in Table S1a in the supplementary material online). Because some PH domains may bind only the plasma membrane, we selected only those PH domain-containing proteins that are known to be endosome related (according to Gene Ontology and UniProt annotation [47,84]). To further identify endosome-associated scaffolds we applied the HPRD, BioGRID, and STRING protein–protein interaction (PPI) resources [46,48,49] to select scaffolds that could bind endosome-related proteins (listed in Table S1b in the supplementary material online). Next, we defined three overlapping classes for the identified 76 endosome-associated scaffolds based on their binding properties (Figure I): (i) scaffolds bind to endosomal membrane; (ii) scaffolds bind to endosome-related proteins; and (iii) indirect endosomal scaffolds bind to directly binding scaffolds (from class ii). Interestingly, only the HGS scaffold has both endosomal membrane and endosome-related protein binding properties. This indicates that HGS has multiple possibilities to bind endosomes and this binding can be highly regulated by the lipid and protein composition of the endosomes.

Finally, using the PathwayLinker web service [85], we examined the pathway memberships of the signaling proteins that interact with endosome-associated scaffolds. PathwayLinker examined the first neighbor interactors (protein partners) of each scaffold based on the interaction data from HPRD, BioGRID, and STRING [46,48,49] and then acquired pathway membership data for the protein partners from the SignaLink, KEGG, and Reactome pathway resources [40,86,87]. The resulting protein–protein interaction network data showing the interactions between the endosomal scaffolds and their partner proteins from different pathways are shown in Table S1c in the supplementary material online). We examined seven biochemically and evolutionarily different pathways (EGF/MAPK, insulin, JAK/STAT, Notch, WNT, TGF-β, Hedgehog) that are known to be important in development and in diseased states [28]. Importantly, crosstalk identification can be possible only if the connecting pathways are well defined. Biochemically based, evolutionarily related pathway grouping (such as presented in [28]) allows crosstalk analysis and avoids artificial pathway constructs and false-positive crosstalk. (Although in the literature the insulin and EGF/MAPK pathways are often separated, according to [28] and with the aim of minimizing false-positive crosstalk in this analysis, we defined the highly overlapping insulin and EGF/MAPK pathways as a single RTK pathway.)

Filtering scaffolds that connect interacting proteins from different pathways allowed us to identify 49 crosstalk-mediating endosomal scaffolds (listed in Table S2a in the supplementary material online). The supporting PPI dataset (Table S2b in the supplementary material online) contains 395 scaffold-mediated crosstalks among 157 signaling proteins. We selected only those crosstalks where the interacting proteins had no mutual pathway memberships to obtain clear crosstalking protein pairs. This was important in the case of interacting multi-pathway proteins (proteins functioning in more than one pathway). Because of this strict filtering, 18 endosomal scaffolds (such as HGS) have been excluded from the short list of 49 clear crosstalk-mediating scaffolds. (The complete list without filtering can be found in Table S1c in the supplementary material online). We note that this is only a list of potential scaffolds capable of mediating crosstalk on endosomes; further in-depth experimental validation is needed to confirm these results.

## Box 3 Technical challenges in studying crosstalk endosomes

Despite the prevalent use of fluorescent techniques to study signaling complexes *in vivo*, there are some major difficulties in studying endosomal signaling and crosstalk [88]. It requires the combined detection of signaling endosome characteristics (such as size, motility, localization, protein, and lipid composition) as well as the analysis of subsequent signaling interactions. Quantitative proteomic studies will be essential to elucidate the full complexity of endosomal compartments. However, the isolation of distinct endosomal populations poses a major challenge. It is also difficult to establish whether signaling complexes localized to endosomes transmit signals or are merely en route to the lysosome for degradation. Furthermore, it is difficult to distinguish signaling events elicited from the plasma membrane from endosome-specific signaling. New microscopy-based fluorescent techniques that can detect signaling molecules in real time are likely to bring us closer to answering the following important questions.

• Can we detect the dynamics and the signaling effect of crosstalk endosomes?
• How are the components of crosstalk endosomes assembled? Is there a coordinated regulatory system to prepare crosstalk endosomes?
• Are there specific populations of crosstalk endosomes? How does the motility and half-life of the endosome and its distance from the nucleus determine its role in signaling?

## Glossary

Chondrogenesis: the process by which cartilage is developed.
Early endosome: the early form of endosomes after the dissociation of the clathrin coat. Early endosomes are enriched in PtdIns(3)P and RAB5 and canonical early endosomes are characterized by the recruitment of early endosome antigen 1 (EEA1), a protein required for homotypic endosomal fusion.
Late endosome: also known as multivesicular bodies (MVBs), they are mainly spherical, lack tubules, and contain many closely packed luminal vesicles. Markers include RAB7, RAB9, and PtdIns(3,5)P_2_.
Phosphoinositides (PtdIns): phospholipids that can be phosphorylated at multiple sites on their inositol ring, this gives rise to various phosphoinositide species. Phosphoinositide kinases and phosphatases reversibly regulate the formation of these species.
Scaffold and adaptor protein: a protein that binds and colocalizes two or more members of a catalytic pathway.
